# Novel patterns of expression and recruitment of new genes on the *t*-haplotype, a mouse selfish chromosome

**DOI:** 10.1098/rspb.2021.1985

**Published:** 2022-02-09

**Authors:** Reka K. Kelemen, Marwan Elkrewi, Anna K. Lindholm, Beatriz Vicoso

**Affiliations:** ^1^ Institute of Science and Technology Austria, Am Campus, 1, 3400 Klosterneuburg, Austria; ^2^ Department of Evolutionary Biology and Environmental Studies, University of Zurich, Winterthurerstrasse, 190, 8057 Zurich, Switzerland

**Keywords:** transmission distortion, gene gain, neofunctionalization

## Abstract

The *t*-haplotype of mice is a classical model for autosomal transmission distortion. A largely non-recombining variant of the proximal region of chromosome 17, it is transmitted to more than 90% of the progeny of heterozygous males through the disabling of sperm carrying a standard chromosome. While extensive genetic and functional work has shed light on individual genes involved in drive, much less is known about the evolution and function of the rest of its hundreds of genes. Here, we characterize the sequence and expression of dozens of *t*-specific transcripts and of their chromosome 17 homologues. Many genes showed reduced expression of the *t*-allele, but an equal number of genes showed increased expression of their *t*-copy, consistent with increased activity or a newly evolved function. Genes on the *t*-haplotype had a significantly higher non-synonymous substitution rate than their homologues on the standard chromosome, with several genes harbouring *dN/dS* ratios above 1. Finally, the *t*-haplotype has acquired at least two genes from other chromosomes, which show high and tissue-specific expression. These results provide a first overview of the gene content of this selfish element, and support a more dynamic evolutionary scenario than expected of a large genomic region with almost no recombination.

## 1. Introduction

Genetic variants that increase their own transmission rate during gametogenesis will spread in the population even if neutral or detrimental with respect to the fitness of the organism [1]. Such transmission distorters, or meiotic drivers, have been found in diverse taxa, including plants, animals and fungi [2,3]. While true meiotic drivers increase their transmission rate by manipulating female meiosis, the so-called ‘sperm killers’ do so by using a poison-antidote system (the ‘driver’ and ‘responder’ genes) to disable sperm not carrying the driver chromosome [4]. Since recombination between the driver and responder genes leads to the creation of suicide chromosomes (which disable all sperm), sperm killers are typically found in regions of no or very low recombination that can harbour large numbers of genes. There has been considerable progress in identifying specific genes underlying the driving mechanisms of different distorters [5–13], but much less is known about how the rest of the gene content of these selfish haplotypes differs from that of their homologous (non-driving) genomic region, and what evolutionary pressures contributed to these changes [7,11,14]. Positive selection will favour mutations that enhance drive, especially if drive-suppressing mutations arise elsewhere in the genome [15]. Such evolutionary arms races can promote the evolution of increasingly complex driving mechanisms involving multiple genes that are co-opted to increase transmission rate [16]. For this reason, many genes linked to the original driving locus may become ‘neofunctionalized’ (i.e. repurposed for segregation distortion). For instance, cooption for drive has been suggested to contribute to the differential expression of large numbers of genes in the testis of stalk-eyed flies carrying a driving X-chromosome [17]. On the other hand, transmission distorters often bear the negative consequences of strong linkage between the driver and responder genes [18]. Reduced recombination between the driving region and its homologous chromosome is often achieved by large inversions, which may trap hundreds of other genes on the driving haplotype [19–22]. These genes are expected to be subject to less efficient purifying selection, which may be compounded if deleterious mutations hitch-hike when new driver mutations sweep to fixation. Genetic degeneration has therefore typically been thought to be the prevalent force shaping gene content on large drivers [14,18,19], although occasional recombination with the non-driving homologue may alleviate this mutation load [23,24].

One of the best-studied autosomal drivers is the *t*-haplotype of house mice, which has served as a model for segregation distortion for nearly 100 years [25,26]. The *t*-haplotype is a sperm killer that achieves above 90% transmission in heterozygous (+/*t*) males, but causes embryonic lethality or adult sterility when present in two copies. A variant form of the proximal half of chromosome 17 thought to have originated more than a million years ago [27,28], it contains four large inversions that link together a region of about 900 genes. Only a few of the genes on the *t*-haplotype have been functionally and evolutionarily characterized, most of these directly related to the driving mechanism. Four genes (*Tagap1, Fgd2, Nme3* and *Tiam2*) have been found to cumulatively distort the transmission ratio [11,29–31], by jointly dysregulating a single target (*Smok1*). The *t*-haplotype codes for an insensitive version of the target (*Tcr*), avoiding the sperm toxicity of *Smok1* overexpression [32]. The fate of the other hundreds of genes originally located on the *t*-haplotype is largely unknown. The drive pathway still has some missing links, and it is thought that the *t*-haplotype probably contains more genes involved in transmission ratio distortion [11], but how many is currently unclear. Interestingly, some of the most differentially expressed genes between carriers and non-carriers of this transmission distorter are on other chromosomes [24,33], but the mechanism underlying this expression upregulation is unknown. Finally, homozygous *t/t* mice typically die as embryos, as most variants of the *t*-haplotype contain recessive lethal mutations [34], but it is unclear whether these are due to widespread degeneration of the whole non-recombining region. While limited evidence of genetic degeneration was detected, this was probably an underestimate, as it was based on short read mapping to the reference, due to the absence of an assembly for the *t*-haplotype [24].

In order to address some of these gaps, we combined published RNA and DNA sequencing data to characterize the sequence and expression evolution of dozens of genes on the *t*-haplotype, and compared their expression and patterns of divergence to those of their homologous chromosome 17 genes. We also describe two highly expressed *t*-specific genes, which were gained from other chromosomes. These results highlight the dynamic evolution of this non-recombining selfish chromosome, at odds with a simple scenario of reduced purifying selection that is expected for a large low recombination region, and potentially suggesting that significant sections of the genome may be co-opted for transmission distortion.

## 2. Results

### (a) Most putative *t*-specific sequences map to chromosome 17

We used published RNA-seq reads obtained from four wild-caught *M. m. domesticus* +/*t* mice (mice heterozygous for the *t*-haplotype [35]) to infer the sequence of genes on the *t*-haplotype. Since these mice also carry one copy of the non-driving chromosome 17, we used three complementary approaches to filter for reads and/or for assembled transcripts that are likely to be *t*-specific (see electronic supplementary material, figure S9): (1) We mapped all RNA-seq reads of +/*t* individuals to the *M. musculus* reference genome, and retained only diverged read pairs (reads with a minimum of three mismatches). We assembled these into transcripts. To detect true *t*-derived sequences, we mapped genomic reads (also from [35]) from 12 +/*t* (*t*-carriers) and 12 +/+ (non-carriers) mice to the assembled transcripts (with no mismatches allowed to avoid cross-mapping with the + allele; see Methods), and selected scaffolds that had a higher genomic coverage (normalized for library size) in all +/*t* mice than in +/+ mice (see electronic supplementary material, data S1). (2) We identified kmers of size 31 that were found in all the RNA and DNA samples of +/*t* mice, but in none of the DNA or RNA samples from +/+ mice, yielding a set of putative *t*-specific kmers. We then selected RNA-seq read pairs from +/*t* samples that contained these *t*-specific 31-mers, and assembled them directly into putative *t*-derived transcripts (see electronic supplementary material, data S2). (3) To complement the assemblies based on pre-filtered reads, we also created an assembly based on all the combined RNA-seq reads derived from all tissues of the four +/*t* mice. The assembled sequences were again filtered based on genomic coverage in 12 +/*t* and 12 +/+ control mice (see electronic supplementary material, data S3). Since this last assembly does not require that reads or transcripts are diverged from the reference, it may include young *t*-specific duplicates.

**Figure 1.**
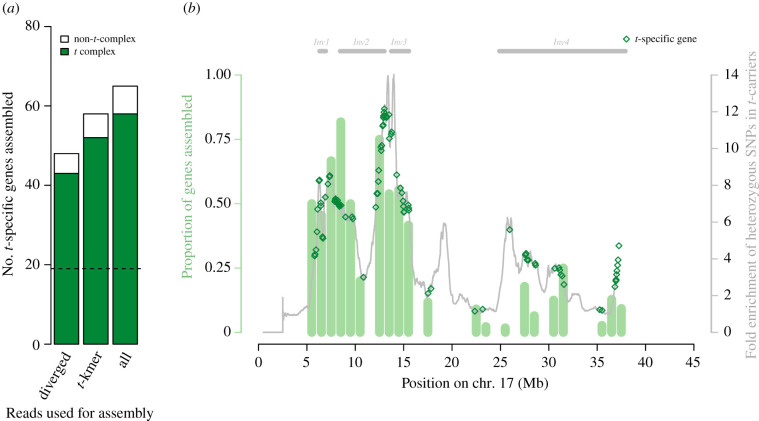
Chromosomal locations of assembled *t*-specific transcripts. (*a*) Numbers of genes for which *t*-specific transcripts were assembled using the three assembly strategies. The proportion of genes mapping to the *t*-complex (3–42 Mb on chromosome 17) is shown in green, while those mapping elsewhere in the genome are in white. The dashed line indicates the number of genes present in all assemblies. (*b*) Proportion and location of genes assembled along the *t*-complex. Light green bars indicate the proportions of genes in 1 Mb windows, for which a *t*-haplotype-specific sequence was assembled. The grey line shows the average excess heterozygosity of *M. m. domesticus* +/*t* mice compared to +/+ mice, adapted from [24]. The locations of *t*-specific genes are shown as green empty diamonds, so mapping genes can be better visualized. The locations of the four inversions along the *t* complex, based on the coordinates of genes confirmed to be in each, are shown on top of the figure.

Transcripts were mapped to the mouse reference genome and transcriptome, and annotated based on which genes they overlapped with (see Methods). More than 90% of our annotated transcripts map to chromosome 17 genes for all three assemblies (figure 1*a*; see electronic supplementary material, data S4 for the annotated list of assembled transcripts), supporting a low false positive rate. Three per cent of all assembled *t*-specific sequences did not map to the mouse reference genome or transcriptome at all (electronic supplementary material, table S1). Forty-five assembled genes are found by at least two assemblies, while 66 genes are detected by a single assembly, showing that the different approaches complement each other well. We find a higher proportion of the genes in the first three inversions of the *t*-haplotype than in the fourth inversion (39–65% versus 5%, *p* < 0.001 with a Fisher’s exact test, figure 1*b*). The fourth inversion is a large paracentric inversion thought to be younger than the second inversion [28], and where *t*-haplotypes are a mosaic of the + and *t*-specific sequences, indicative of recombination events [24,36]. The greater level of divergence between the *t* and the standard chromosome in the proximal half of the *t* complex probably gave us more power to assemble *t*-specific transcripts from this region.

### (b) Decreased and increased expression of *t*-specific alleles are equally common

We investigated patterns of expression of *t*-derived transcripts in eight tissues obtained from four +/*t* mice and four +/+ mice of the subspecies *M. m. domesticus* [35] (see electronic supplementary material, figure S10A). We used Kallisto [37], a software suitable for inferring allele-specific expression, to estimate transcript abundance of both putative *t*-transcripts and of their chromosome 17 homologues. We tested our power to infer *t*-specific expression by simulating reads from the sequence of both the *t* and the + alleles, and re-estimating expression levels with the simulated reads. The simulated ratio of expression between the two homologues was recovered by Kallisto for all but one gene (*Mup9*), which we excluded from further analysis (see electronic supplementary material, figure S1). Only transcripts that produced an alignment longer than 300 base pairs with a + transcript in the *t* complex, and for which average expression was >1 transcripts per million (TPM) for at least one tissue, were kept for further analysis (58 out of 111 putative *t*-specific genes; see electronic supplementary material, data S5).

**Figure 2.**
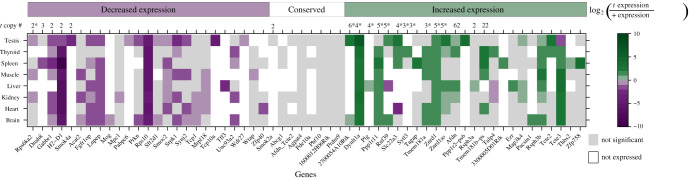
Relative expression of *t*-specific alleles in the *t* complex. Colour coding shows the log_2_ ratio of the average expression of the *t* allele in +/*t* mice to that of the + allele in +/ + mice. Non-significant differences in expression are coloured grey, while tissues with no expression (on average < 1 TPM for both alleles) are white. Genes whose *t* allele is underexpressed (purple) in at least one tissue and do not show overexpression (green) in any tissue are shown on the left. Genes with conserved *t* allele expression in all tissues with expression are shown in the middle. Genes whose *t* allele is overexpressed in at least one tissue are shown on the right. The numbers of gene copy gain detected in 4 +/*t* versus 4 +/ + *M .m. domesticus* DNA samples are indicated on top of the figure, with asterisks denoting fixed copy gain among the four +/*t* mice.

In order to understand changes in gene expression that have arisen specifically on the *t*-haplotype, we compared the expression of *t* transcripts to the expression of the + allele in +/+ mice. As a control, we also compared the expression of the + allele between +/*t* and +/+ mice. The (misassigned) expression level of the *t* allele in +/+ mice was used to correct the TPM of the + and *t* alleles in +/*t* mice (see Methods). Overall, the *t* allele deviated significantly in expression for 51% of the tissue comparisons, while the + allele deviated only for 14% of such comparisons (*p* < 0.0001, Fisher’s exact test). We classified each *t* allele into one of three categories based on its expression: (1) conserved expression, if there was no significant difference (with a Wilcoxon test) between the expression of the *t* allele and the + allele in any tissue; (2) decreased expression, if the *t* allele had a significantly lower expression compared to the + allele in at least one tissue, and was conserved otherwise; (3) increased expression, if the *t* allele had a significantly higher expression compared with the + allele in at least one tissue, which might be a sign of increased activity or a newly acquired function in the tissue(s). While 25 genes were underexpressed on the *t*-haplotype (left side of figure 2), another 25 genes were overexpressed in at least one tissue on the *t*-haplotype (right side of figure 2). Eight genes, shown in the middle of figure 2, have conserved expression of the *t* allele in all tissues where the gene is expressed. Applying no correction for the fraction of TPM misassigned between alleles changed the classification of only one gene in the degeneration group and one gene in the conservation group (electronic supplementary material, figure S2). Comparing the *t* allele’s expression against the + allele’s expression within +/*t* mice changed the classification of 14 individual genes, but led to similar patterns of over- versus underexpression (electronic supplementary material, figure S3).

We detected no dependence between the overexpression of + alleles and the underexpression of *t* alleles (*p* = 0.08, binomial test, electronic supplementary material, figure S4), indicating that our allele-specific expression estimation is not systematically biased towards one allele. Genes overexpressed on the *t*-haplotype are enriched for copy gain events (taken from [24]) that are either polymorphic or shared among the four +/*t* mice when compared to genes in the decreased expression (Fisher’s exact test, *p* = 0.004 and *p* = 0.002, respectively) or conservation (Fisher’s exact test, *p* = 0.02 and *p* = 0.03, respectively) groups, suggesting that gene amplification may be an important mechanism through which expression is modulated [24]. Varying polymorphism levels among the gained copies suggest different ages of amplification (electronic supplementary material, figure S5).

### (c) Faster accumulation of non-synonymous mutations on the *t*-haplotype

**Figure 3.**
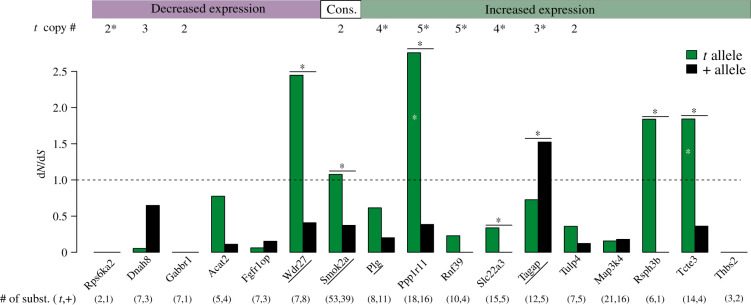
Ratios of non-synonymous to synonymous substitution rates (*dN/dS*) of *t* (green) and + (black) alleles of 16 *t* complex genes. Only genes with a CDS alignment of at least 100 base pairs and d*S* > 0 on both the + and *t* lineages were included. Black stars on top of the bars mean that d*N*/d*S* is significantly different between the *t* and + alleles, and white stars indicate d*N*/d*S* values significantly higher than 1 (using likelihood ratio tests; see Methods). The estimated mean copy number gained by 4 +/*t* *M. m. domesticus* mice is indicated on top of the figure, with asterisks denoting fixed copy gain among the four +/*t* mice. Underlined genes have a premature STOP codon in their *t* alleles. The numbers of substitutions in the *t* and + alleles are shown in parentheses on the bottom (for numbers of synonymous and non-synonymous substitutions see electronic supplementary material, data S7). The boxes on top of the figure indicate the *t* allele’s expression pattern (figure 2). (Online version in colour.)

Both reduced purifying selection and increased positive selection can lead to increases in the rate of protein coding divergence. We therefore tested if *t*-haplotype genes show an elevated ratio of non-synonymous to synonymous substitution rates (*dN/dS*) when compared with their freely recombining homologues. We selected *t*-specific genes that had a coding sequence (CDS) alignment of at least 100 base pairs with the + allele, and that had annotated homologues in the sister species, the Algerian mouse (*M. spretus*), and the distant relative, the rat (*R. norvegicus*) (35 genes; see electronic supplementary material, data S7 and figure S10B). We concatenated alignments of these genes, excluding four genes that had premature STOP codons (underlined genes names in figure 3), and estimated d*N*/d*S* for all branches using the software PAML [38]. The branch leading to the *t*-haplotype had a *dN/dS* two-fold higher than the rest of the branches (*p* < 0.0001; likelihood-ratio-test of models with branch-specific or shared d*N*/d*S* values), generally consistent with what is observed in regions of low recombination due to the accumulation of deleterious mutations [18,39,40].

We also plotted *dN/dS* values for the *t*-haplotype and *M. musculus* (+) branches for all individual genes that had a rate of synonymous substitution (*dS*) greater than 0 in both lineages (figure 3). Six genes show a significantly higher *dN/dS* on the *t*-haplotype than on the + allele. Two genes (*Ppp1r11* and *Tcte3*) have *dN*/*dS* values significantly higher than one, suggesting that these genes may have undergone positive selection after becoming part of the *t*-haplotype.

### (d) The *t*-haplotype expresses modified copies of genes gained from other chromosomes

**Figure 4.**
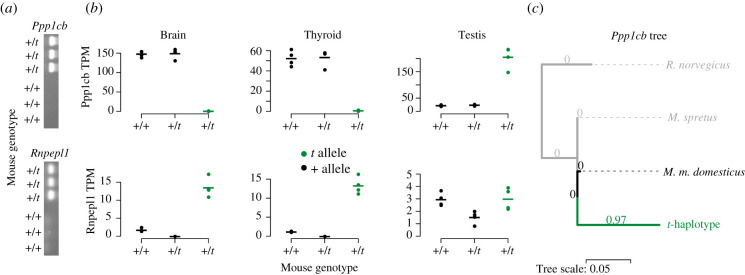
Presence, expression and sequence evolution of gained genes on the *t*-haplotype. (*a*) PCR bands showing the presence of the *t*-specific copies of *Ppp1cb* and *Rnpepl1* in 3 +/*t* mice and their absence in 3 +/+ mice (for all 20 mice tested, see electronic supplementary material, table S2). (*b*) Expression in the three tissues, where the gained genes are differentially expressed [24]. Dots show Transcripts Per Million (TPM) measured in individual mice, while the horizontal bars show the average of the four mice. Expression is shown in green for the *t*-specific copy and in black for the paralogues on the other chromosomes. (*c*) Phylogenetic tree estimated by PAML based on the sequence alignment of *Ppp1cb*. The ratio of non-synonymous and synonymous substitution rates, *dN/dS,* was estimated for each branch separately, as this model was superior to one with shared d*N*/d*S* (*p* < 0.0001, likelihood ratio test). *dN/dS* values are shown above each branch. (Online version in colour.)

Our set of candidate *t*-specific sequences included copies of eight genes, which are located outside of chromosome 17 (one gene each from chromosomes 1, 2, 4, 5, 6, 15 and three genes from chromosome 16). The majority has very low absolute expression, or low expression relative to the parental copy (electronic supplementary material, figure S7). However, two genes, *Rnpepl1* and *Ppp1cb* showed high expression, and had previously been found to be strongly overexpressed in *t*-carrier mice [24,33]. It had been suggested that functional elements on the *t*-haplotype might be regulating these genes in *trans* [24,33]. However, patterns of genomic read coverage of +/*t* and +/+ samples (electronic supplementary material, figure S6) strongly support the presence of a copy of these genes on the *t*-haplotype itself. PCR amplification of these sequences yielded strong bands in all 10 +/*t* mice tested, and no or very faint bands in +/+ mice (figure 4*a*; electronic supplementary material, figure S8 and table S2), confirming the presence of a *t* copy of these genes.

*Rnpepl1* is overexpressed in the brains and thyroid glands, while *Ppp1cb* is overexpressed in the testes of *t*-carrier mice [24,33] (figure 4*b*). The current analysis shows that, for both genes, overexpression comes from the *t*-specific paralogue, with the parental copy being expressed at similar levels in +/*t* and +/+ mice (figure 4*b*). In the case of *Rnpepl1*, the *t*-haplotype expresses a nonsense-mediated decay copy of the gene, which contains only an 80-amino-acid-long truncated version of the protein. The *t*-specific paralogue of *Ppp1cb* expresses a putative protein-coding transcript at a 10-fold higher level in the testis than the chromosome 5 paralogue. Contrary to the original *Ppp1cb*, the *t*-specific paralogue is not expressed in any other tissue. We aligned the *t*-specific *Ppp1cb* sequence to that of the paralogue in *M. m. domesticus* and the orthologues in *M. spretus* and *R. norvegicus*, and estimated non-synonymous and synonymous substitution rates using PAML. While *Ppp1cb* is generally highly conserved, without a single non-synonymous mutation detected on any of the non-*t* lineages, the paralogue on the *t*-haplotype differs by 20 non-synonymous substitutions, resulting in a *dN/dS* of 0.97 (figure 4*c*).

## 3. Discussion

The *t*-haplotype has been a model for meiotic drive for nearly a century. While a lot is known about the molecular mechanism and the key genes used for achieving drive, studying the entire sequence of the *t*-haplotype has not yet been possible. Here we performed a partial characterization of the gene content of the *t*-haplotype by assembling *t*-specific transcripts from RNA-seq reads, and assessing their expression and sequence evolution. Of the 878 genes of the *t* complex, we assembled 111 genes. Since only data from +/*t* mice was available, we were limited to regions of the *t*-haplotype that were differentiated in sequence from the homologous chromosome 17 regions and/or duplicated on the *t*-haplotype, thus yielding increased genomic read coverage in +/*t* mice compared to +/ + mice. The average divergence of assembled *t*-specific sequences was 0.022. Owing to our genomic-coverage-based selection of *t*-specific sequences, involving samples from three subspecies, we are unable to recover genes specific to certain *t* variants. Although we probably underestimate degeneration by missing unexpressed or deleted genes, copy number estimation [24] showed that there are only four genes in the *t* complex that overlap a deletion fixed among *M. m. domesticus* +/*t* mice. Furthermore, significant underexpression in +/*t* mice compared to +/+ individuals affects only a minority of *t* complex genes (4 and 77 genes, in [24,33], respectively). Although gene expression buffering/dosage compensation from the standard chromosome could mask the underexpression of degenerated genes on the *t*-haplotype, we find no correlation between the underexpression of *t* alleles and the overexpression of + alleles in this study. This suggests that degeneration due to deletions or lack of expression from *t* alleles is fairly limited. On the other hand, since only a minority of chromosome 17 genes are differentially expressed between +/*t* and +/ + individuals (21 and 195 in [24,33], respectively), and there are signs of widespread recombination between the *t*-haplotype and the standard chromosome 17 [24], a large proportion of the genes on the *t*-haplotype are probably undifferentiated in both sequence and expression (and therefore missed here).

While our study is restricted to diverged genes, it provides a first overview of the dynamic evolution of the gene content and expression of this large transmission distorter. In total, 43% of the *t*-specific genes in our expression analysis are overexpressed in at least one tissue when compared to the + allele. While the accumulation of neutral or deleterious mutations in regulatory regions could also lead to increased expression [41], this raises the interesting possibility that some may have acquired new functions since becoming part of the *t*-haplotype. Although no functional enrichment can be found after correcting for multiple comparisons, several of these genes with upregulated expression have a functional annotation related to plasma membrane bounded cell projection, such as sperm flagellum, cilium, microvillus and microspike (7 out of 25 genes; *p* = 0.0018 without correcting for multiple testing, see electronic supplementary material, table S3), making it plausible that they are involved in drive. However, differential expression of *t*-specific genes is not limited to the testis, and it is possible that some of these differentially expressed genes give the *t*-haplotype a selective advantage without direct involvement in sperm function. For example, +/*t* mice show behavioural differences compared to +/+ mice, such as increased aggression in males [42] or higher likelihood to disperse from their populations [43,44], both of which have been hypothesized to facilitate the spread of this transmission distorter.

Our results also show that, contrary to a model of simple degeneration, selfish elements can gain genes from other chromosomes, similar to the gain of genes by non-recombining Y chromosomes [45,46]. While functional studies are needed to infer the role of the new copies of *Rnpepl1* and *Ppp1cb*, their high and tissue-specific expression suggests a possible contribution to the biology of the *t*-haplotype. The overexpression of the *t*-specific paralogue of *Rnpepl1*, an aminopeptidase, in the brain makes it an interesting candidate for the behavioural differences associated with +/*t* mice, but the lack of a substantial open reading frame supports at most a regulatory function. On the other hand, the *t*-specific paralogue of the protein phosphatase *Ppp1cb* shows signs of very fast protein evolution and is highly and exclusively expressed in the testes of *t*-carriers. Protein phosphatase 1 complexes are important for spermatogenesis, with one of the active forms suppressing sperm motility in the epididymis [47,48]. It is therefore possible that the new copy of *Ppp1cb* is involved in the drive exhibited by the *t*-haplotype. The fact that two other *t*-complex PPP1-related genes (*Ppp1r11* and *Ppp1r2ps6*) show highly increased expression of their *t*-derived transcripts in the testis, and that *Ppp1r11*’s rate of non-synonymous substitution is suggestive of positive selection, provides further support for the role of these proteins in the biology of the *t*-haplotype.

Genome and transcriptome assemblies of large transmission distorters coupled with allele-specific expression and sequence evolution analysis have the prospect of showing how degenerate selfish haplotypes are and of uncovering driver-specific functionality [18]. Future genomic assemblies that include the entire *t*-haplotype will reveal the full extent of conservation and divergence in sequence and expression on this classic model for transmission distortion.

## 4. Methods

For a detailed description of the methods and scripts, see the electronic supplementary material, methods. Pipelines are shown in electronic supplementary material, figures S9 and S10.

### (a) Assembling diverged reads

We pooled RNA-seq reads from 10 tissues sampled from four *M. m. domesticus* mice heterozygous for the *t*-haplotype [35] (https://www.ebi.ac.uk/ena/browser/view/PRJEB9450). We trimmed the first and last five base pairs off of every read using a custom perl script. Trimmomatic [49] (version 0.38, with parameters LEADING:20 TRAILING:20 SLIDINGWINDOW:4:25 MINLEN:36) was used to remove bases with quality below 20 at the beginning and end of reads, windows of 4 base pairs with an average base quality below 25, and Illumina adapters. Reads shorter than 36 base pairs after trimming were removed. To select diverged reads, we mapped trimmed RNA-seq reads to the GRCm38.p6 genome using Tophat [50] (v. 2.1.1 with default settings). Reads with more than two mismatches were unmapped, and all paired unmapped reads were assembled into scaffolds using Trinity [51] (v. 2.12.0 with default parameters).

### (b) Assembling reads with *t*-specific kmers

We used genomic libraries of four +/*t* and four +/+ *M. m. domesticus* mice as well as transcriptomic libraries from these mice with up to 10 tissues pooled per mouse [35] (https://www.ebi.ac.uk/ena/browser/view/PRJEB9450). Following [52], we used the script kcompress.sh in the software BBMap [53] to output the unique 31 base pair kmers in each of the four +/*t* genomic libraries and each of the four +/*t* RNA-seq libraries. We found 31-mers shared between all +/*t* 31-mer sets, by setting the mincount parameter to 8 in the script kmercountexact.sh. We then removed any 31-mer present in any of the four genomic or RNA-seq libraries of the +/+ control mice using bbduk.sh. We recovered RNA-seq reads from *t*-carrier libraries that overlapped in at least 30% of their lengths with *t*-carrier specific kmers, by setting the ‘minkmerfraction’ parameter to 0.3 in bbduk.sh. The recovered reads from the four *t*-carrier mice were pooled and assembled using Trinity, as before.

### (c) Assembling unfiltered reads

Pooled, untrimmed and unfiltered RNA-seq reads from up to 10 tissues of four *M. m. domesticus* +/*t* mice, were assembled into scaffolds with the software Trinity (default parameters).

### (d) Filtering based on genomic reads

We masked repetitive sequences in our assembled sequences with RepeatMasker [54] (using the combined database Dfam 3.1 and rmblastn v. 2.10.0+), and filtered for a minimum unmasked length of 300 base pairs. We mapped the first read in each pair of genomic reads in 12 carrier and 12 non-carrier samples to the sequences with Bowtie2 [55] (v. 2.3.4.1 with default parameters). We filtered for a higher abundance of perfectly matching reads (normalized for library size) in all +/*t* samples than in +/+ samples.

### (e) Annotation of assembled sequences

We mapped RepeatMasker-masked sequences against the GRCm38.p6 genome and transcriptome using BLAT [56] (v. 35x1 with parameters −*t* = dnax − *q* = dnax). Sequences that overlapped multiple neighboring genes were further examined based on CDS overlap and assigned to a single gene whenever possible (see electronic supplementary material, methods).

### (f) Gene-specific re-assembly and sequence selection

We grouped sequences by gene annotation from the divergence-based and kmer-based assemblies together and from the unfiltered-reads-based assembly separately, and re-assembled scaffolds into longer sequences using the software Cap3 [57] (version 02/10/15, with a maximum overhang of 80% and requiring at least 40% overlap of at least one scaffold).

### (g) Expression estimation

We aligned *t* sequences to GRC38.p6 transcripts using BLAT (version 35x1 with parameters −*t* = dnax − *q* = dnax), and for each gene we retained the longest alignments (minimum 300 base pairs). The assembly of unfiltered reads was only used when genes were not found in the other assemblies. For all other genes, the longest transcripts were included. We used RNA-seq libraries from four +/*t* and four +/+ *M. m. domesticus* mice obtained from eight tissues [35] (electronic supplementary material, figure S9A). We trimmed reads using Trimmomatic, and estimated expression levels of *t* and + transcripts from each sample using the software Kallisto [37] (v. 0.46.2 with default parameters). Transcript abundance estimates were normalized by library size. Genes with average expression below 1 TPM in all individuals for both the *t* and + transcripts were removed from the analysis.

### (h) Correcting for ambiguity in read assignment

In R [58] (v. 3.6.3) we calculated the proportion of ambiguity in +/+ samples by dividing the average TPM mis-assigned to the *t* allele by the average total TPM assigned to that gene. In each sample, we subtracted this proportion from both the *t*-transcript’s and the + transcript’s TPM values.

### (i) Testing for differential expression

For each gene and tissue, we used a Wilcoxon signed rank test (in R) on the four corrected expression values of the *t* transcript in +/*t* mice and the four corrected expression values of the + transcript in +/+ mice divided by two.

### (j) Simulating reads for testing the expression estimation of Kallisto

Using the software ART [59] (v. 2.5.8) we generated Illumina Hiseq 2000 paired-end reads (91 base pairs, standard deviation of 10, fragment size of 180 base pairs, mimicking our real reads) from all the *t* and + transcripts that were included in our expression analysis. Expression estimation was the same as on the real dataset.

### (k) Coding sequence alignments

We aligned each *t* transcript to the + peptide sequences of the corresponding gene using the software GeneWise [60] (v. 2.4.1 with default settings), and retained the translated *t* peptide with the longest alignment, if it was longer than 100 base pairs. We used the *t* peptide sequence to align the *M. musculus* + transcript, as well as the *R. norvegicus* and *M. spretus* orthologous transcripts (obtained from the ensembl database BioMart [61] (release 104)) to it using GeneWise. For genes with orthologues in both species CDS alignments were made using TranslatorX [62] (v. 1.1 with default settings).

### (l) Estimating *dN*/*dS*

We used the *codeml* function of PAML [38] (v. 4.9j) to estimate *dN/dS* from alignments. We used the species tree as the input tree (see electronic supplementary material, methods). To test if the total *dN/dS* on the *t*-haplotype is larger than that on other lineages we compared a null model of shared *dN/dS* among all lineages (model = 0) and an alternative model of only the *t*-haplotype having its own *dN/dS* value (model = 2 and a distinct branch label on the input tree). To test if a single gene has different d*N*/d*S* values on the *t*-haplotype and on the + chromosome, we compared a null model of shared d*N*/d*S* of these two lineages and an alternative model of distinct d*N*/d*S* values. To test if a gene has a d*N*/d*S* value above 1, the null model was the site-branch model with *ω*_2_ fixed at 1 (model = 2, NSites = 2, fixomega = 1, omega = 1), and the alternative model was the full site-branch model (model = 2, NSites = 2, fixomega = 0, omega = 2).

### (m) Statistical comparison of different PAML models

We extracted log-likelihood (lnL) estimates of PAML, and calculated the Akaike information criterion (AIC) score for each model using the formula 2k-2lnL, where k is the number of parameters (d*N*/d*S* values estimated) in a model. AIC score differences above 2 units were considered to be significant.

### (n) Finding genes overlapping copy number variant regions in *t*-carrier mice

We used copy number variants (CNVs) called by the software Control-FREEC [63] (version 10.5 with parameter window = 1000 or 5000) for the four *M. m. domesticus* +/*t* mice and a pool of four +/ + mice as controls (same as in [24]). With BEDTools’ *intersect* function [64] we found genes overlapping CNVs, and we averaged the estimated copy number inferred in the 1 kb and 5 kb windows for each gene in R.

### (o) Primer design and PCR

We designed two primer pairs each for *Ppp1cb* and *Rnpepl1* (see electronic supplementary material, methods) that contained *t*-specific mutations at their 3’ ends, using the software Primer3 and its default settings [65] (version 0.4.0). The primers were tested on an independent set of *M. m. domesticus* (see [66] for population details; study design and sampling procedures were approved by the Veterinary Office, Zurich Switzerland (permit 215/2006)). All mice were genotyped using the *Hba4-ps4* and *Vil2* primers, which produce bands of different sizes in +/+ and +/*t* mice. We first ran the PCR with the first set of primers per gene, on only three +/+ and three +/*t* mice (shown in figure 4 and electronic supplementary material, figure S8). To confirm these results, we conducted PCR on another 10 +/+ and another 10 +/*t* mice (summarized in electronic supplementary material, table S2). We isolated DNA using salt–chloroform extraction [67]. We used PCR conditions of 94° for 7 min, and 32 cycles of 94° for 30s, 60° for 60s and 72° for 120 s and then a 20 min extension at 72°. We ran the samples on a 1% agarose gel. We analysed PCR products using a 3730xl DNA Analyzer (Applied Biosystems) and Genemapper software (Applied Biosystems).

### (p) Gene ontology enrichment analysis

We used the MouseMine website with default settings and no test correction to find enrichment in the ‘cellular component’ ontology.

## Data accessibility

This article has no additional data.
